# Synthesis of Nanocrystal-Embedded Bulk Metallic Glass Composites by a Combination of Mechanical Alloying and Vacuum Hot Pressing

**DOI:** 10.3390/ma18020360

**Published:** 2025-01-14

**Authors:** Pee-Yew Lee, Pei-Jung Chang, Chin-Yi Chen, Chung-Kwei Lin

**Affiliations:** 1 Department of Optoelectronics and Materials Technology, National Taiwan Ocean University, Keelung 202-301, Taiwan; pylee@ntou.edu.tw; 2 Research Center of Digital Oral Science and Technology, College of Oral Medicine, Taipei Medical University, Taipei 110-301, Taiwan; peronchang@tmu.edu.tw (P.-J.C.); chencyi@fcu.edu.tw (C.-Y.C.); 3 Graduate Institute of Manufacturing Technology, National Taipei University of Technology, Taipei 106-344, Taiwan; 4 Department of Materials Science and Engineering, Feng Chia University, Taichung 407-102, Taiwan; 5 School of Dental Technology, College of Oral Medicine, Taipei Medical University, Taipei 110-301, Taiwan

**Keywords:** mechanical alloying, amorphization, vacuum hot pressing, bulk metallic glass, nanocrystals

## Abstract

Bulk metallic glasses (i.e., BMGs) have attracted a lot of research and development interest due to their unique properties. Embedding BMG composites with nanocrystals can further extend their applications. In this study, Ta-nanocrystal-embedded metallic glass powder was prepared via the mechanical alloying of (Cu_60_Zr_30_Ti_10_)_91_Ta_9_ composition for 5 h using starting elemental powders. The structural evolution during the mechanical alloying process was examined using X-ray diffraction, scanning electron microscopy, synchrotron extended X-ray absorption fine structure, transmission electron microscopy, and differential scanning calorimetry. The 5 h as-milled powder was then consolidated into a bulk sample using vacuum hot pressing with an applied pressure of 0.72, 0.96, and 1.20 GPa. The effects of the applied pressure during vacuum hot pressing on the structure of the obtained BMG were investigated. The experimental results show that Ta-nanocrystal-embedded metallic glass composite powder was prepared successfully after 5 h of mechanical alloying. The 5 h as-milled composite powder exhibited a large supercooled region of 43 K between the glass transition temperature of 743 K and the crystallization temperature of 786 K. Using vacuum hot pressing at 753 K for 30 mins with an applied pressure, dense nanocrystal-embedded BMG composites were synthesized. The relative density and the crystallization temperature of the BMG composites increased with increasing applied pressure. The nanocrystal-embedded BMG composites prepared at 753 K for 30 mins with an applied pressure of 1.20 GPa exhibited a relative density of 98.3% and a crystallization temperature of 786 K. These nanocrystals were Ta, Cu_51_Zr_14_, and other possible Cu–Zr–Ti alloys (e.g., Cu_10_Zr_7_) that were randomly dispersed within the glassy matrix.

## 1. Introduction

Metallic glass has been an attractive advanced engineering material for the past few decades. It is generally formed using rapid cooling, with a cooling rate larger than 100 K/s, followed by solidification [1]. Due to its unique structure and excellent properties, metallic glass has been widely used in many applications [2]. Another important achievement was presented by Inoue et al. in the 1980s that allowed for further innovation in metallic glass production. For instance, Pd [3], Zr [4], La [5], Mg [6], and Fe [7] based BMGs have been investigated and manufactured with a thickness larger than 10 mm and a cooling rate as low as 1 K/s. This extends the practical applications of BMGs [8]. Recently, growing research and development activities in the additive manufacturing technology further stimulate the research and development of BMGs. For instance, bulk BMGs with complex geometry and functional gradients can be prepared via additive manufacturing technologies [9,10,11].

Examples of attractive BMG systems are Cu-based bulk metallic glasses that exhibit a high tensile strength exceeding 2000 MPa, a large critical diameter of 28.5 mm, and a relatively low cost compared to other alloy systems [12]. Cu-based alloy systems, including Cu–Hf–Ti, Cu–Ti–Zr, Cu–Ti–Zr–Ni–Si, Cu–Ti–Zr–Ni–Sn, Cu–Zr–Hf–Ti, and Cu–Zr–Ti–Y, have been investigated [13,14,15]. A series of ternary Cu–Zr–Ti BMGs were designed, and the corresponding mechanical properties were examined [16]. Recently, Cu-based BMG without rare-earth and precious metal addition was synthesized and exhibited excellent glass-forming ability and processability. In particular, Cu_46_Zr_33.5_Hf_13.5_Al_7_ exhibited the best glass-forming ability, with a critical casting thickness of 28.5 mm, which is the largest among all Cu-based BMGs [12]. The crystallization of this unique metallic glass was investigated to better understand its large-glass-forming ability and thermal stability [17,18]. To further enhance the performance of these bulk glassy alloys, Cu-based bulk metallic composites were investigated [19,20,21,22]. For instance, Cu–Zr–Ti BMG composites were obtained by mixing various compositions of Cu_50_Zr_50_ and Cu_73_Ti_27_ binary alloys, followed by suction casting [23]. The addition of Ta in the Cu_0.50_Hf_0.35_Ti_0.10_Ag_0.05_ BMGs increased not only their compressive fracture strength but also their elastic–plastic strain [19]. It is also reported that the mixed structure of the glassy and second phase can significantly increase the plastic elongation and mechanical strength of the (Cu_0.6_Hf_0.25_Ti_0.15_)_94_Ta_6_ composite material [22]. In addition, Cu_41_Ni_27_Ti_25_Al_7_ BMG composite with excellent plasticity and a strong work-hardening behavior was fabricated [24]. By using spark plasma sintering, high-strength and high-conductivity bulk CuCrZr alloy/CuZrAl metallic glass composites were prepared [25]. Meanwhile, the utilization of controlled atomic diffusion using cryo-cooled copper-mold suction casting enabled the preparation of phase-separated Cu_46_Zr_46_Al_8_ bulk metallic glass (BMG) and had the potential to be used for design optimization [26].

Typically, most BMGs and their composites are prepared using conventional copper mold casting with rapid cooling. Spark plasma sintering [25], die casting [27], and additive manufacturing [9,10,11] can also be used to prepare the BMGs. In addition, hot pressing of Cu-based amorphous alloy powder prepared by gas atomization was also used to successfully prepare BMGs for mechanical properties examination [28,29]. However, the large discrepancy between the melting temperatures of the constituents makes the melting and casting processes difficult, which may result in residues of the unmelted refractory elements or their early precipitation from the melt. In addition, downhill segregation of the refractory constituents may occur due to their relatively high densities. For instance, Ta (16.6 g/cm^3^) has a higher density than that of Cu_60_Zr_30_Ti_10_ BMGs (7.4 g/cm^3^) [30]. A substitute way to subdue these difficulties is to first synthesize BMG powders by mechanical alloying and then consolidate them into bulk metallic glass. Originally developed by Benjamin, who used the mechanical alloying (MA) process to synthesize strengthened superalloys with uniformly distributed oxide particles [31], the high-energy ball milling process has been adopted to prepare numerous metastable materials, including amorphous powders and composites. For instance, MA has been successfully used to prepare Mg- [6], Zr- [32], and Cu-based [33,34] glassy composite powders. By milling a mixture of ductile Cu, Zr, and Ti elements, repetitive cold welding, deformation, and fracturing can occur during the mechanical alloying process. This leads to continuous refinement and interdiffusion of starting elements, resulting in amorphization [34]. By adding another brittle component (like WC powder) into the system, ductile metallic elements (i.e., Cu, Zr, and Ti) undergo an amorphization process, whereas brittle particles are cracked into small particles and embedded within an amorphous matrix [35].

Mechanical alloying of Cu, Zr, Ti, and Ta ductile powder mixture may differ from the abovementioned Cu, Zr, Ti, and WC system. With the addition of a refractory Ta element for which the amount can be easily adjusted, Ta is expected to be refined and dispersed uniformly within the amorphous matrix during the mechanical alloying process. If followed by vacuum hot pressing of the as-milled powder, bulk composite samples can be prepared for desired property evaluations before utilizing them in practical applications. This powder metallurgical approach to preparing refractory metal nanocrystal-embedded bulk metallic glass composites is seldom reported. This approach allows for numerous metallic glass systems to be investigated, while also allowing autonomous selection of the amount and type of refractory metal element to be added to the BMG composite based on application requirements. This study uses Ta as the refractory element addition, and (Cu_60_Zr_30_Ti_10_)_91_Ta_9_ was selected as the prototype. The feasibility of preparing nanocrystal-embedded bulk metallic glass composite using mechanical alloying of (Cu_60_Zr_30_Ti_10_)_91_Ta_9_ composition followed by vacuum hot pressing was evaluated. The structural evolution during mechanical alloying and the effect of applied pressure during vacuum hot pressing for the preparation of nanocrystal-embedded metallic glass powder and composites were investigated.

## 2. Materials and Methods

### 2.1. Mechanical Alloying and Characterization of (Cu_60_Zr_30_Ti_10_)_91_Ta_9_ Composition

Cu (99.9%, <300 mesh), Ti (99.9%, <100 mesh), Zr (99.9%, <325 mesh), and Ta (99.8%, <100 mesh) powders were weighed according to the designed (Cu_60_Zr_30_Ti_10_)_91_Ta_9_ composition. A total of 4 g of powder mixture and ~20 g of 7 mm Cr–steel balls (ball to powder = 5:1) were put into an SKH 9 high-speed steel vial with a diameter and height of 40 mm and 50 mm, respectively. Within an argon-filled glove box, a SPEX 8016 shaker ball mill (Fisher Scientific, Ottawa, ON, Canada) was operated at 1725 rpm and used for mechanical alloying treatment. All the canning and milling processes proceeded within the glove box where the oxygen and humility were controlled to less than 1 ppm to minimize oxidation. During the procedures, the milling process was interrupted every 30 min and stopped for 15 min to cool down the vial. An appropriate amount of powder after milling for various times was taken out for required characterizations.

The structural evolution during the high-energy ball milling process was examined using X-ray diffraction (XRD), scanning electron microscopy (SEM), synchrotron extended X-ray absorption fine structure (EXAFS), transmission electron microscopy (TEM), and differential scanning calorimetry (DSC). The XRD examination was performed by using a Bruker X-ray diffractometer (AXS GmbH-D2 PHASER, Billerica, MA, USA) with Cu Kα emission that was filtered by a Ni thin film. The obtained XRD patterns were analyzed further by the XRD analysis software EVA (version 5.2, Brucker-AXS EVA, Brucker, WI, USA) to determine the crystalline phases, whereas the grain size of the selected sample was calculated by using Scherrer’s formula with a shape factor (k) equaling 0.9. The powder morphology and cross-sectional observation of powder after selected milling times were examined using a field emission scanning electron microscope (Hitachi S-4800, Hitachi, Tokyo, Japan) that was equipped with energy-dispersive X-ray (EDX) microanalysis. The as-milled powders were directly dispersed on carbon conductive tape for morphological observation. For cross-sectional views, the powders were cold mounted with epoxy resin, ground by sandpapers, and polished by alumina nanopowder. The etching solution consisting of 60 mL glycerin, 15 mL hydrofluoric acid, and 15 mL nitric acid was used to etch the polished sample. Gold sputtering was used to improve the conductivity of samples for SEM observation.

Synchrotron extended X-ray absorption fine structure (EXAFS) measurements were executed at NSRRC beamline (Wiggler-17C, National Synchrotron Radiation Research Center, Hsinchu, Taiwan). The nanocrystalline and amorphous phases within the 5 h as-milled powder were investigated using a JEOL JEM-1400 transmission electron microscope (JEOL Ltd., Tokyo, Japan). A small amount of powder (~0.1 wt.%) was dispersed in alcohol by an ultrasonic bath. A few droplets of the top layered solution were dropped on a copper grid with a formvar carbon film. The so-obtained sample was then placed in a vacuum furnace set at 80 °C for 12 h before TEM observation. Selected-area electron diffraction (SAED) was performed at areas of interest. In addition, by using the Image J software (Fiji Image J2, version 1.54f, National Institutes of Health, Bethesda, MD, USA), d spacing from various diffraction rings and spots was obtained and used to determine the crystalline phases. In addition, the glass transition and crystallization temperatures of amorphous powder were measured by using a Dupont 2000 differential scanning calorimeter (DSC, TA instruments, New Castle, PA, USA) at a heating rate of 40 K/min with a continuous flow of 140 cc/min high-purity argon gas. A small amount of as-milled powder (~10 mg) was placed in a copper holder and an empty one was used as a reference for the DSC measurement.

### 2.2. Consolidation and Examination of 5 H As-Milled (Cu_60_Zr_30_Ti_10_)_91_Ta_9_ Powder

The 5 h as-milled powders were consolidated by vacuum hot pressing at 753 K (determined by the DSC result) for 30 min with an applied pressure of 0.72, 0.96, and 1.20 GPa, respectively. High-purity argon gas was used to purge the system before vacuum, and the heating rate for vacuum hot pressing was set at 40 K/min (same as the DSC test). After consolidation, the hot-pressed samples were examined using the aforementioned XRD, DSC, and TEM techniques to reveal the materials’ characteristics. For TEM examination, the hot-pressed sample was sliced into a thin layer with a size of 5 × 5 mm^2^ and polished continuously to a thickness of less than 20 μm. Then, an ion beam miller was employed to gradually remove the surface at an incident angle of ~6 ^o^ to obtain the sample for TEM observation. In addition, Archimedes’ method was used to determine the relative density of the hot-pressed samples (*n* = 3) prepared at different applied pressures.

## 3. Results and Discussion

### 3.1. Preparation of Ta-Nanocrystal-Embedded Metallic Glass Composites by Mechanical Alloying

In order to elucidate the structural evolution of mechanical alloying (Cu_60_Zr_30_Ti_10_)_91_Ta_9_ powder mixture during high-energy ball milling treatment, X-ray diffraction, SEM observation, synchrotron extended X-ray absorption fine structure (EXAFS), TEM investigation, and differential scanning calorimetry were used to examine the as-milled powder at various milling stages.

**Figure 1:**
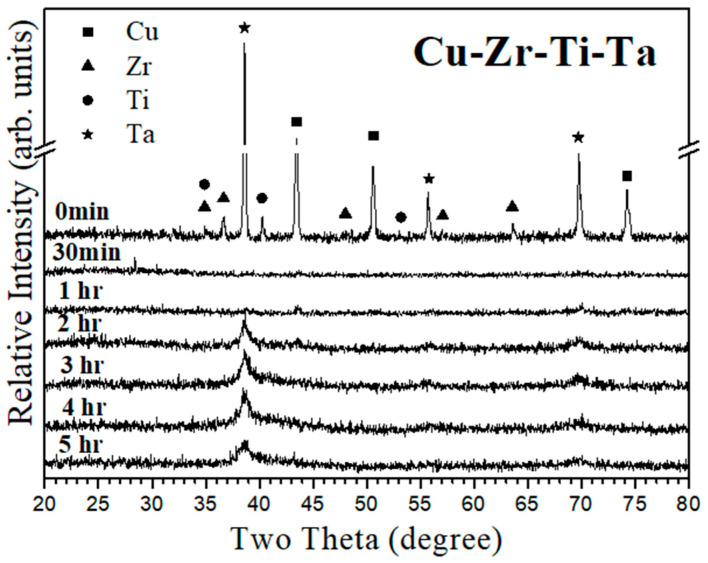
X-ray diffraction patterns of (Cu_60_Zr_30_Ti_10_)_91_Ta_9_ powders as a function of milling time.

Figure 1 shows a series of X-ray diffraction patterns of original and as-milled (Cu_60_Zr_30_Ti_10_)_91_Ta_9_ powder at various stages of mechanical alloying. At the beginning of milling (the top XRD pattern in Figure 1), crystalline peaks from the starting powder mixture were clearly observed. However, they quickly disappeared after 30 min of mechanical alloying treatment. An individual XRD pattern of 30 min as-milled powder is shown in Figure S1, where an ambiguous amorphous peak at a relatively low angle (~25°) was observed. A similar diffraction pattern was observed for 1 h as-milled powder. This shows a similar result for mechanical alloying of Cu_60_Zr_30_Ti_10_, where limited diffraction peaks were noticed after 1 h of milling treatment [34]. When milling time was prolonged to 2 h, the as-milled powder exhibited broadened diffraction peaks from elemental tantalum, and similar diffraction patterns were observed thereafter. As reported in the literature [34], mechanical alloying of Cu_60_Zr_30_Ti_10_ resulted in amorphous metallic glass powder after 5 h of milling treatment. In this study, the 5 h as-milled (Cu_60_Zr_30_Ti_10_)_91_Ta_9_ powder did not exhibit a distinct amorphous representing broadening peak, which may be superimposed by the relatively strong diffraction peaks from Ta nanocrystals. The crystalline size of Ta (BCC structure, PDF# 04-0788) after 5 h of milling was 16.21 ± 3.84 nm, calculated using Scherrer’s formula. In addition, it should be pointed out that the major diffraction peak of the starting Ta powder was 38.47°, slightly shifted to a higher angle (38.84°) after 5 h of milling. The calculated lattice constant was 0.331 and 0.328 nm for starting and 5 h as-milled powder, respectively. This can be attributed to the partial replacement of Ta by Cu (possessing an atomic size smaller than that of Ta) and results in a shrinkage of unit cell. This suggests that Ta (or limited Ta solid solution) nanocrystals formed after mechanical alloying treatment.

**Figure 2:**
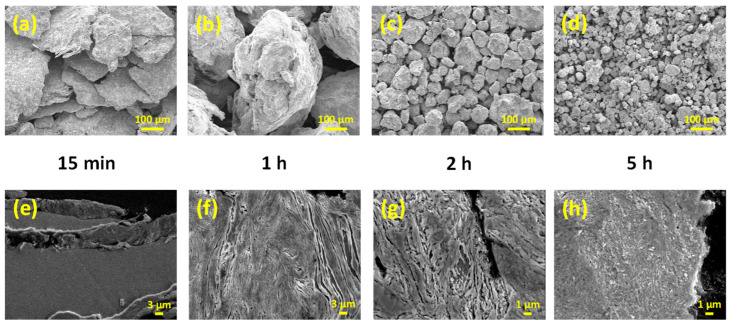
SEM images of (Cu_60_Zr_30_Ti_10_)_91_Ta_9_ powder after different milling times (15 min, 1 h, 2 h, and 5 h). The images in upper row (**a**–**d**) and lower row (**e**–**h**) are powder morphologies and cross-sectional views, respectively.

Scanning electron microscopy was used to observe the powder morphology and examine the cross-sectional images of selected as-milled powder. As shown in Figure 2a, the 15 min as-milled powder was plate-like. Since all the starting elemental powder (Cu, Zr, Ti, and Ta) was ductile, the plate-like particles indicated severe cold welding at the early stage of milling. When milling time increased to 1 h, repetitive cold welding, fracture, and agglomeration of the as-milled powder resulted in relatively large particles (Figure 2b). Thereafter, continuous refinement of the as-milled powder was observed (2 h and 5 h as-milled powder, Figure 2c and Figure 2d, respectively). The cross-sectional views were examined to further resolve the mechanical alloying process. The starting powder became lamellar and cold welded together during the high-energy ball milling. Relatively thick lamellae were exhibited after a short milling time (15 min as-milled, Figure 2e). Continuously refined lamellae were observed for 1 h and 2 h as-milled powder (Figure 2f,g, respectively). Alloying occurred due to the interdiffusion of starting elements and resulted in a homogeneous microstructure after 5 h of milling (Figure 2h).

**Figure 3:**
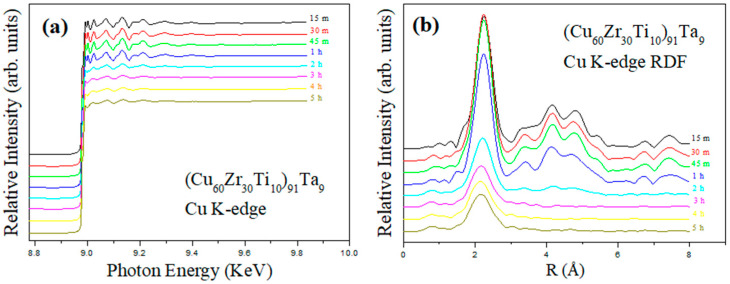
(**a**) Extended X-ray absorption spectra at Cu K edge and (**b**) radial distribution functions of Cu atom as a function of milling time.

The X-ray diffraction investigated the long order of crystalline structure, whereas synchrotron EXAFS examination revealed the short-range atomic variation of the as-milled powders during mechanical alloying. Figure 3a shows the EXAFS spectra of the Cu K edge (i.e., 8979 eV) at various milling times. It can be observed that the amplitude of fluctuation decreased with the increasing milling time. This suggests that the crystallinity of the Cu elements decreased with prolonged milling time. After Fourier transforming the EXAFS spectra into radial distribution function (RDF), the local environment of Cu atoms can be determined [36]. Figure 3b shows the corresponding RDFs of the as-milled powders. It can be observed that the magnitudes of the nearest neighbor contributors decreased slightly at the early stage of milling (say within 1 h). Significant decreases in peaks’ magnitudes (especially high-order peaks) were observed for 2 h as-milled powder. After 3 h of milling, a slight decrease in the distance of the nearest neighbor (determined by the peak position) can be observed. No significant difference can be observed thereafter. This suggests that the Cu element began to amorphize with other elements after 2 h of milling treatment and exhibited no significant difference after 3 h of milling. This suggests that the amorphous phases became dominant after 3 h of milling. Similar observations have been reported in various systems, such as Fe–Ta, Mg–Y–Cu, and Ni–Zr–Ti–Si [36,37,38].

**Figure 4:**
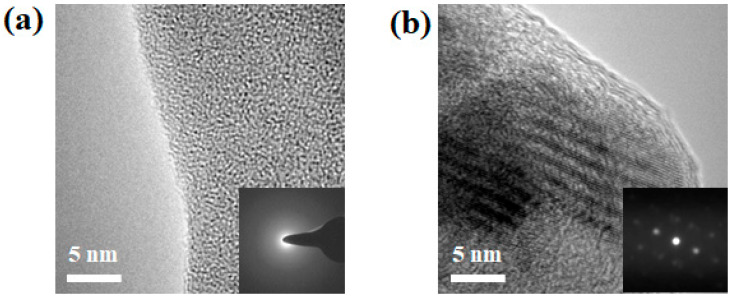
TEM images of (**a**) amorphous matrix and (**b**) Ta nanocrystals after 5 h of mechanical alloying treatment. The inserts on the bottom-right corners are the corresponding selected-area electron diffraction patterns.

Transmission electron microscopy (TEM) was used to confirm the final microstructure of 5 h as-milled (Cu_60_Zr_30_Ti_10_)_91_Ta_9_ powder. Figure 4a shows the bright-field TEM image of the matrix that exhibited a classical salt and pepper image, suggesting the formation of a homogeneous amorphous phase. The selected-area electron diffraction (SAED) pattern, shown in the insert in the bottom right corner, exhibited an amorphous-representing diffuse halo ring. This confirms the formation of an amorphous metallic glass matrix, though the amorphous peak was absent in the X-ray diffraction pattern (Figure 1). Meanwhile, it was observed that some nanocrystals were uniformly dispersed in the amorphous matrix. Figure 4b shows the TEM image of two overlapped Ta nanocrystals (the black fringed area) with a grain size of 14.18 ± 2.86 nm, similar to that (16.21 ± 3.84 nm) estimated by XRD peak broadening. The corresponding SAED pattern was as shown in the insert figure and identified as a Ta nano-sized particle (Figure S2). The calculated lattice constant of Ta nanocrystal was 3.28 ± 0.05 nm, slightly smaller than the original Ta element (3.30 nm). This confirms the XRD results as shown in Figure 1, in which diffraction peaks from Ta elements persisted or formed a limited solid solution after 5 h of mechanical alloying treatment. TEM observation revealed that the 5 h as-milled (Cu_60_Zr_30_Ti_10_)_91_Ta_9_ powder consisted of a metallic glass matrix embedded with Ta nanocrystals. A combination of X-ray diffraction (a relatively large amount of powder was examined) and TEM observation (only localized areas were examined) suggests that 9 at.% of Ta nanocrystals (or a limited Ta solid solution phase) uniformly dispersed in an amorphous Cu_60_Zr_30_Ti_10_ metallic glass matrix after 5 h of mechanical alloying treatment.

**Figure 5:**
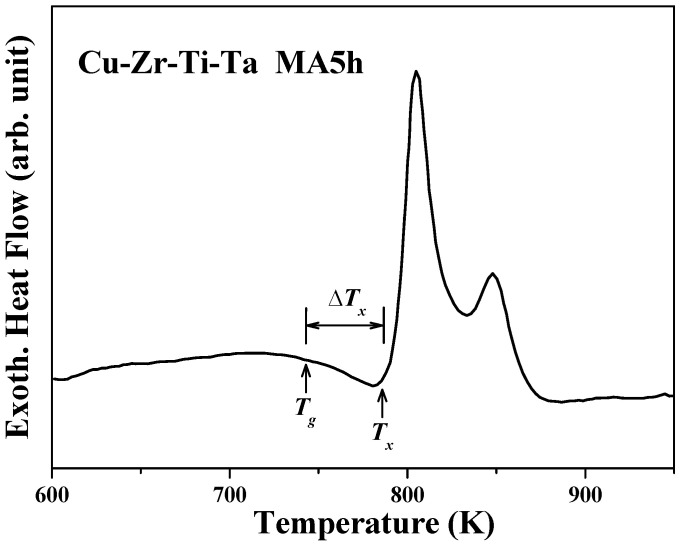
DSC curve of 5 h as-milled (Cu_60_Zr_30_Ti_10_)_91_Ta_9_ powder.

As demonstrated above by the XRD, SEM, EXAFS, and TEM characterizations, composite powders consisting of Ta nanocrystals embedded in the metallic glass matrix were synthesized with 5 h of MA treatment. The 5 h as-milled composite powder was used as the starting material to prepare nanocrystal-embedded bulk metallic glass composite and the resulting composite’s thermal property was examined using differential scanning calorimetry to determine the appropriate temperature for vacuum hot pressing. Figure 5 shows the DSC curve of 5 h as-milled (Cu_60_Zr_30_Ti_10_)_91_Ta_9_ powder. An endothermic event indicated that glass transition was observed at 743 K and was followed by a distinct exothermic crystallization peak at 786 K, revealing a sequential transformation from a supercooled liquid to crystallinities. Thus, the supercooled liquid region (ΔTx) was 43 K for 5 h as-milled composite powder. It should be pointed out that the DSC curve was measured at a heating rate of 40 K/min (same as the heating rate of vacuum hot pressing), and a higher heating rate shifted the onset temperatures toward higher temperatures.

### 3.2. Consolidation and Characterization of Nanocrystal-Embedded BMG Composites

**Figure 6:**
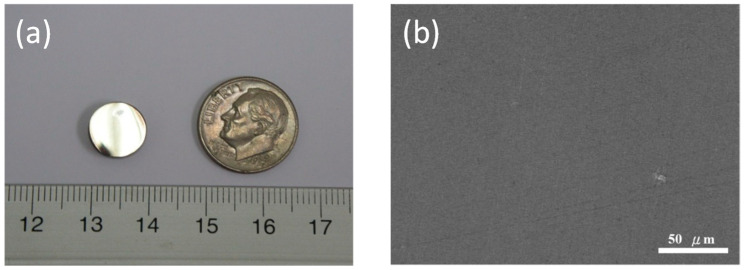
(**a**) Photo and (**b**) polished cross-sectional view of a composite sample prepared at a pressure of 1.20 GPa. The unit of the ruler in Figure 6a is centimeters.

Though all of the milling processes were performed under an argon-filled glove box and the vacuum hot-pressing system was purged with high-purity argon gas, hundreds to thousands ppm of oxygen depending on the purity of starting elemental powders was inevitable and may affect the performance of BMGs [32,39]. The 5 h as-milled powders were vacuum hot pressed at 753 K (10 K above T_x_) for 30 min with an applied pressure of either 0.72, 0.96, or 1.20 GPa. Figure 6a shows a photo of a typical consolidated composite sample prepared at a pressure of 1.20 Gpa. It exhibited a smooth outer surface and metallic luster. Figure 6b shows the polished cross-sectional view of the BMG composite sample where only limited pores were observable in the SEM image. The relative density for composite samples (*n* = 3) prepared at 0.72, 0.96, and 1.20 GPa was 96.7 ± 0.3%, 97.5 ± 0.3%, and 98.3 ± 0.2%, respectively. The higher the applied pressure during vacuum hot pressing, the higher the density of the composites. Due to the limitation of the equipment and safety concerns, 100% dense BMG composites were not achieved. The results, however, suggest that a highly dense bulk (Cu_60_Zr_30_Ti_10_)_91_Ta_9_ composite was prepared successfully by vacuum hot pressing.

**Figure 7:**
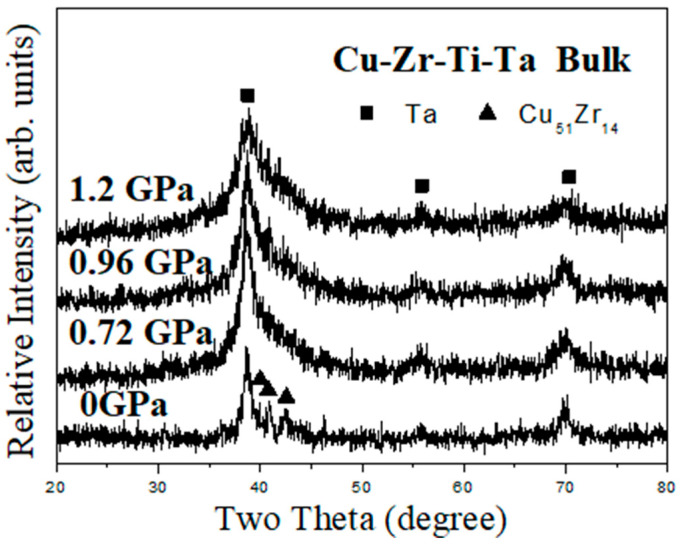
XRD spectra of the composites were prepared by vacuum hot pressing at 753 K for 30 min with or without an applied pressure.

The crystalline structure of the hot-pressed bulk composite samples was examined using X-ray diffraction. Figure 7 shows the XRD spectra of the composites prepared by vacuum hot pressing at 753 K for 30 min with or without an applied pressure. As shown in the bottom XRD pattern in Figure 7, it can be observed that partial crystallization of the starting amorphous matrix occurred and formation of Cu_51_Zr_14_ (hexagonal structure, PDF# 42-1185), Cu_10_Zr_7_ (hexagonal structure, PDF# 47-1028), or other Cu–Zr–Ti phases was observed. Figure S3 shows the analysis results by Rietveld’s fitting method. With an applied pressure of 0.72, 0.96, or 1.20 GPa, similar XRD patterns were obtained. The top three XRD patterns shown in Figure 7 exhibited an amorphous-representing broad diffraction peak and three Ta diffraction peaks at 2θ = 39.01°, 56.01°, and 70.18°. Compared to the starting material (5 h as-milled powder, bottom XRD pattern in Figure 1), an amorphous broad diffraction peak can be observed after hot pressing. This can be attributed to the stress relaxation and recovery of the severe plastic deformation induced by mechanical alloying. In addition, it is interesting to note that the Ta diffraction peak at 39.01° became ambiguous with increasing applied pressure. This suggests that vacuum hot pressing with a high applied pressure can suppress the formation of Cu_51_Zr_14_ or other Cu–Zr–Ti phases.

**Figure 8:**
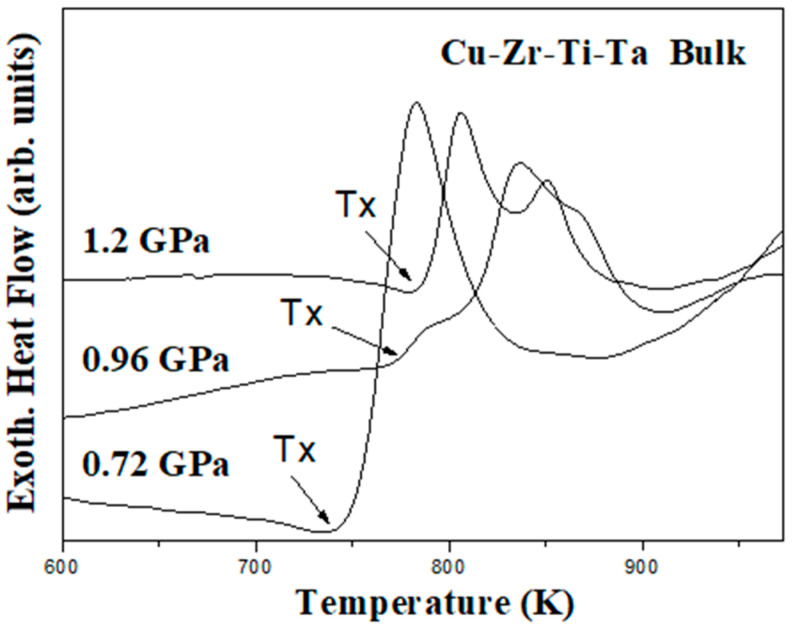
DSC curves of nanocrystal-embedded bulk metallic glass composites prepared at 753 K for 30 min with an applied pressure of 0.72, 0.96, and 1.20 GPa, respectively.

Figure 8 shows the DSC traces of (Cu_60_Zr_30_Ti_10_)_91_Ta_9_ BMG composites consolidated at different pressures. It can be observed that all the DSC traces of hot-pressed samples exhibited an exothermic characteristic of a crystallization reaction during the continuous heating process. This indicates that the primary structure of all hot-pressed samples was still amorphous. In addition, it was observed that the crystallization temperature (T_x_) of the hot-pressed samples increased with increasing applied pressure. The crystallization temperature was 743, 776, and 786 K for BMG composite prepared with an applied pressure of 0.72, 0.96, and 1.20 GPa, respectively. A similar phenomenon has been reported in Zr-based BMGs and amorphous semiconductors [40,41]. Compared to the DSC curve of 5 h as-milled composite powder (Figure 5), BMG composite prepared using 1.20 GPa possessed the same crystallization temperature. When combined with the XRD analysis addressed above in Figure 7, this confirms that the crystallization reaction was confined during vacuum hot pressing with an applied pressure of 1.20 GPa.

The crystallization behaviors of BMGs under an applied pressure have been investigated comprehensively [41,42]. Generally, three different effects may occur for the preparation of BMG with an applied pressure [43]. Firstly, the closest structure is preferred during densification and the crystallization process. Secondly, at high pressures, atomic mobility is suppressed, and the atomic diffusion in metallic glasses is reduced. Thirdly, the relative Gibbs free energies of the glassy and crystalline phases and the energy barrier for crystallization change under the applied pressure. Both the second and third effects may dominate the crystallization behavior during the vacuum hot pressing process. By investigating the crystallization of glassy alloys, Jiang et al. [44] considered that metallic glass crystallization is a process that proceeds by the nucleation and successive growth of crystals. Atomic diffusion and volume change affect the crystallization at the initial nucleation stage within the metallic glass. The activation energy barriers for nucleation and diffusion will govern the crystallization temperature. The nucleation rate, I, can be written as I = I_0_/exp [(ΔG* + Q_n_)/k_B_T], where I_0_ is a constant, ΔG* is the free energy for the formation of a nucleus with the critical size, Q_n_ is the activation energy for the transportation of an atom from the interface to the nucleus, and k_B_ is Boltzmann’s constant. The required nucleation work is the summation of ΔG* and Q_n_. The Pd–Ni–P bulk glass crystallizes via a eutectic reaction, wherein the atoms will be rearranged during the nucleation process, and the pressure dependence of Q_n_ might be a dominant factor. The atomic mobility will decrease with an applied pressure, suggesting an increase in Q_n_ and the corresponding nucleation work. This results in a decrease in nucleation rate and an increase in the crystallization temperature.

**Figure 9:**
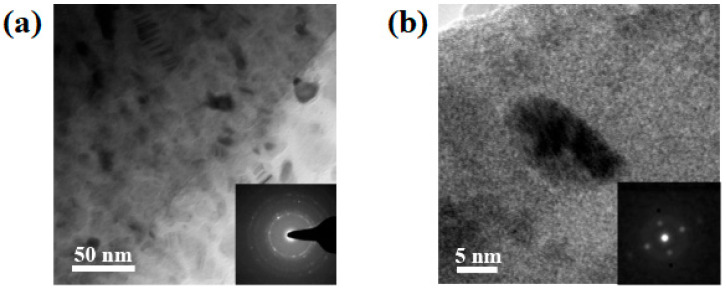
(**a**) TEM images of nanocrystal-embedded bulk metallic glass composite prepared at 753 K for 30 min with an applied pressure of 1.20 GPa. The insert shows the Cu_51_Zr_14_ and Cu_10_Zr_7_ nanocrystals. (**b**) Magnified TEM image shows a Ta nanocrystal that was confirmed by the SAED pattern in the insert.

Recently, the crystallization of Cu_50_Zr_40_Ti_10_ amorphous powder was investigated by Cai et al. [45]. The crystallization of Cu_10_Zr_7_ phase in either isothermal or non-isothermal conditions was observed for amorphous powders prepared via gas atomization and mechanical milling. In addition, Cai et al. also examined the preparation and crystallization of planetary ball-milled plate-like Cu–Zr–Ti amorphous alloy composite powders [46]. Amorphization sequence and crystallization behavior in both non-isothermal and isothermal crystallization modes were discussed. For Cu_70_Zr_15_Ti_15_ composition, crystalline Cu_3_Ti and Cu phases were observed for isothermal crystallization, whereas Cu_51_Zr_14_, Cu_3_Ti, and Cu formed for non-isothermal crystallization. Instead of planetary ball milling, high-energy shaker milling was used in the present study and amorphization via mechanical alloying was induced by repetitive cold welding, fracturing, and interdiffusion of starting elements. After 5 h of MA treatment, highly deformed particles and a large number of dislocations were exhibited within the amorphous composite powder and served as nuclei for crystallization. The competing results of three pressure effects promote the formation of more crystalline nuclei, while the atomic mobility of long-range diffusion is simultaneously suppressed at high pressures. As shown by the XRD and DSC results (Figure 7 and Figure 8), crystallization was more evident without applied pressure or relatively low applied pressure. This suggests that crystallization was restrained due to suppressed atomic mobility under high applied pressure (e.g., 1.20 GPa). With increasing temperature and time, crystalline nuclei grew within the hot-pressed sample. Eventually, the crystalline nuclei tend to form nanocrystals embedded within the metallic glass matrix. A more detailed analysis of the XRD pattern for the 0 GPa hot-pressed sample was performed using the Rietveld fitting method. Figure S3 shows that it exhibited a best fit of Ta, Cu_51_Zr_14_, and possible Cu_10_Zr_7_ (or other Cu–Zr–Ti alloys) phases within the sample. This suggests that, in addition to the pre-existing Ta nanocrystals that formed after 5 h mechanical alloying treatment, Cu_51_Zr_14_ together with Cu_10_Zr_7_ or other Cu–Zr–Ti alloys were the preferred phases during crystallization. Though only Ta peaks were observed clearly for the BMG composites prepared by using an applied pressure (top three curves in Figure 7), the formation of Cu_51_Zr_14_ and other Cu–Zr–Ti alloys (e.g., Cu_10_Zr_7_) nanocrystals was possible. TEM observation was used to confirm this phenomenon, and Figure 9a shows the TEM image of a hot-pressed sample prepared using an applied pressure of 1.20 GPa. The sizes of the nanocrystalline particles ranged from 10 to 20 nm. The phase of crystalline precipitations was identified as Cu_51_Zr_14_ and Ta by using the corresponding selected-area electron diffraction pattern shown in the insert at the bottom-right corner. The SAED pattern was analyzed by using the Image J software and is shown in Figure S4a. A magnified TEM image of the hot-pressed sample is shown in Figure 9b, where the insert at the bottom-right corner was identified as Ta nanocrystals (Figure S4b) that resulted after 5 h milling treatment. The TEM observations show that hot-pressed samples resulted in nanocrystal-embedded bulk metallic glass composite that has nanocrystals formed from randomly distributed Ta, Cu_51_Zr_14_, and other possible Cu–Zr–Ti alloys (e.g., Cu_10_Zr_7_) within the metallic glass matrix. Similar observations have been observed in other BMG composites [46,47,48].

Though the present work mainly focused on the preparation of nanocrystal-embedded BMG composites, preliminary examination of the hot-pressed BMG composites showed that the Vickers microhardness increased with increasing applied pressure. It was 509 and 614 kg/mm^2^ for samples prepared with an applied pressure of 0.72 and 1.20 GPa, respectively. These BMG composites with tunable composition of metallic glass matrix and adjustable amount of refractory metal addition were potential candidates for functional and structural applications. Further application-oriented characterizations, including thermal stability, mechanical and antibacterial properties, corrosion resistance at various solutions, etc., are in progress and will be addressed elsewhere.

## 4. Conclusions

In the present study, mechanical alloying technique was used to successfully prepare Ta nanocrystal-embedded metallic glass composite powder after 5 h of milling treatment. The 5 h as-milled composite powders exhibited a T_g_ and T_x_ at 743 K and 786 K, respectively, which resulted in a large supercooled region (ΔT_x_) of 43 K. After the consolidation of 5 h as-milled composite powder using vacuum hot pressing with an applied pressure, relatively dense nanocrystal-embedded BMG composites were obtained. The relative density was 96.7%, 97.5%, and 98.3% for BMG composites prepared at 0.72, 0.96, and 1.20 GPa, respectively. In addition, the crystallization temperature (T_x_) of the BMG composites increased with increasing applied pressure and was 743, 776, and 786 K for 0.72, 0.96, and 1.20 GPa, respectively. BMG composites consisted of randomly distributed nanocrystals within the metallic glass matrix. These nanocrystals consisted of Cu_51_Zr_14_ and possible Cu_10_Zr_7_ (or other Cu–Zr–Ti alloys) phases that were formed during vacuum hot pressing and elemental Ta (or a limited Ta solid solution phase) that was continuously refined after 5 h of milling treatment. By using the powder metallurgy methods (mechanical alloying followed by vacuum hot pressing), nanocrystal-embedded Cu-based bulk metallic glass composites were successfully prepared. Thus, their material properties can be evaluated before practical application.

## Data Availability

The original contributions presented in this study are included in the article and Supplementary Materials. Further inquiries can be directed to the corresponding author.

## Supplementary Materials

The following supporting information can be downloaded at: https://www.mdpi.com/article/10.3390/ma18020360/s1, Figure S1: X-ray diffraction pattern of (Cu_60_Zr_30_Ti_10_)_91_Ta_9_ powder after 30 mins of milling. Figure S2: Magnified SAED pattern with indexes for Ta nanocrystal within 5h as-milled powder. Figure S3: X-ray diffraction pattern of the composite prepared by vacuum hot pressing at 753K for 30 min without applied pressure. Please note that only three major peaks for each phase were shown to avoid confusion. Figure S4: Magnified SAED patterns with indexes for (a) Cu_51_Zr_14_ (dash yellow curves) and Ta (dash white curve) nanocrystals and (b) Ta nanocrystal for hot-pressed BMG composite prepared with an applied pressure of 1.20 GPa.
