# Supplementary material for: Synthesis of Nanocrystal-Embedded Bulk Metallic Glass Composites by a Combination of Mechanical Alloying and Vacuum Hot Pressing
Source: Materials (Basel). 2025 Jan 14;18(2):360. doi: 10.3390/ma18020360 (PMC11766767; doi:10.3390/ma18020360)
Supplement: Supplementary file 1 [file materials-18-00360-s001.zip › materials-3339390-supplementary.pdf]

Figure S1 shows the magnified X-ray diffraction patterns (the XRD pattern from Fig. 1) of $(Cu_{60}Zr_{30}Ti_{10})_{91}Ta_9$ powder mixture after 30 mins. of milling. An ambiguous amorphous peak can be noticed.

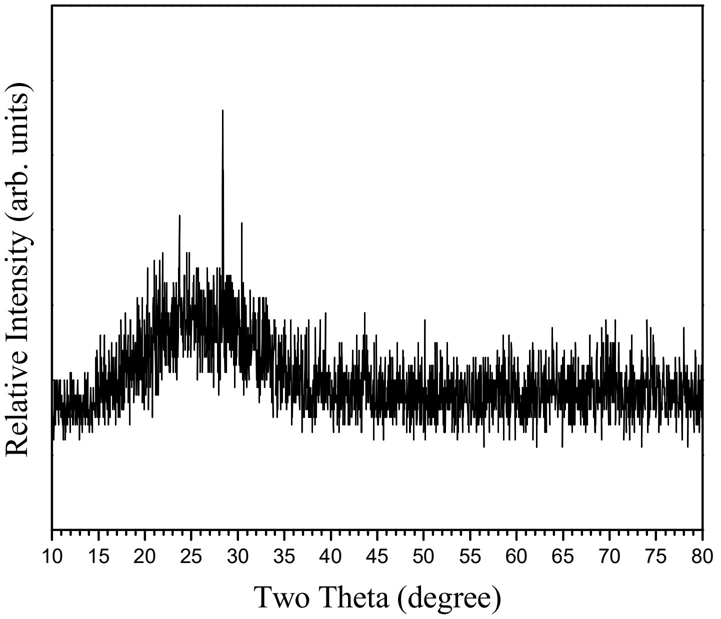

Figure S1. X-ray diffraction pattern of $(Cu_{60}Zr_{30}Ti_{10})_{91}Ta_9$ powder after 30 mins. of milling.

Figure S2 shows the analysis result of the SAED pattern for 5h as-milled powder. The calculated lattice constant of Ta nanocrystal was 3.28 ± 0.05 nm, which is only slightly smaller than the original Ta element (3.30 nm).

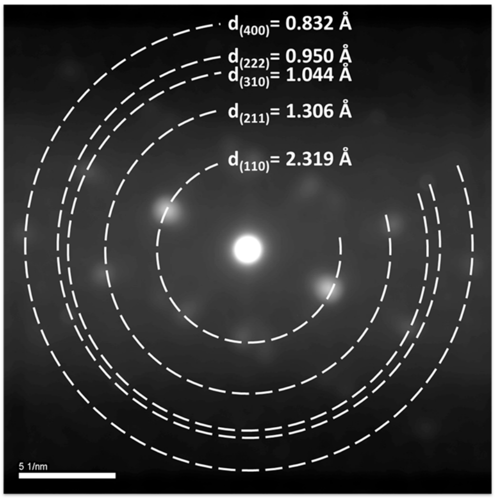

Figure S2. Magnified SAED pattern with indexes for Ta nanocrystal within 5h as-milled powder.

Figure S3 shows the X-ray diffraction pattern of the hot-pressed sample prepared by vacuum hot pressing at 753K for 30 min without applied pressure. By using the Rietveld fitting method, the XRD pattern exhibited the best fit of Ta, $Cu_{51}Zr_{14}$, and possible $Cu_{10}Zr_7$ phases. However, other Cu-Zr-Ti phases may be exhibited within the hot-pressed sample.

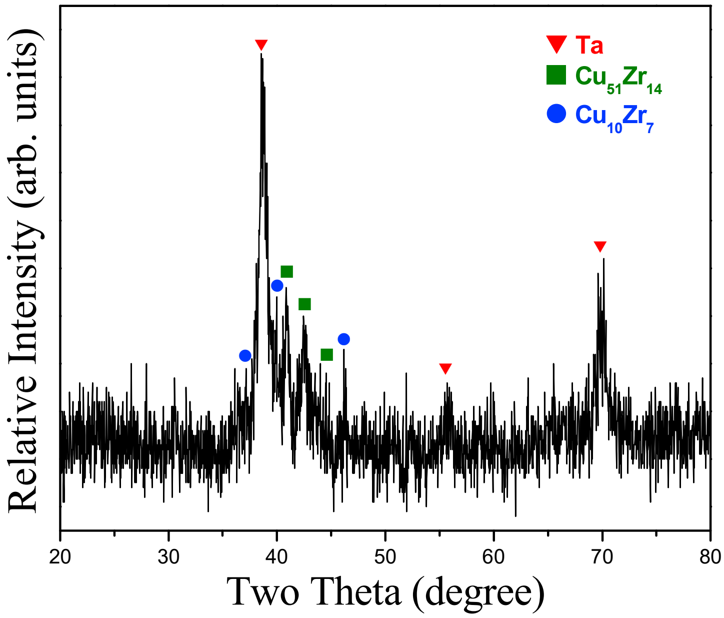

Figure S3. X-ray diffraction pattern of the composite prepared by vacuum hot pressing at 753K for 30 min without applied pressure. Please note that only three major peaks for each phase were shown to avoid confusion.

Figure S4 shows the analysis result of the SAED patterns from two selected regions of 1.20 GPa hot-pressed BMG composite corresponding to those shown as inserts in Figs. 9a and 9b where $Cu_{51}Zr_{14}$ and Ta nanocrystals were identified. The calculated lattice constant of Ta nanocrystal was 3.29 ± 0.01 nm, not significantly different from the original Ta element (3.30 nm) or that within 5h as-milled powder (3.28 nm).

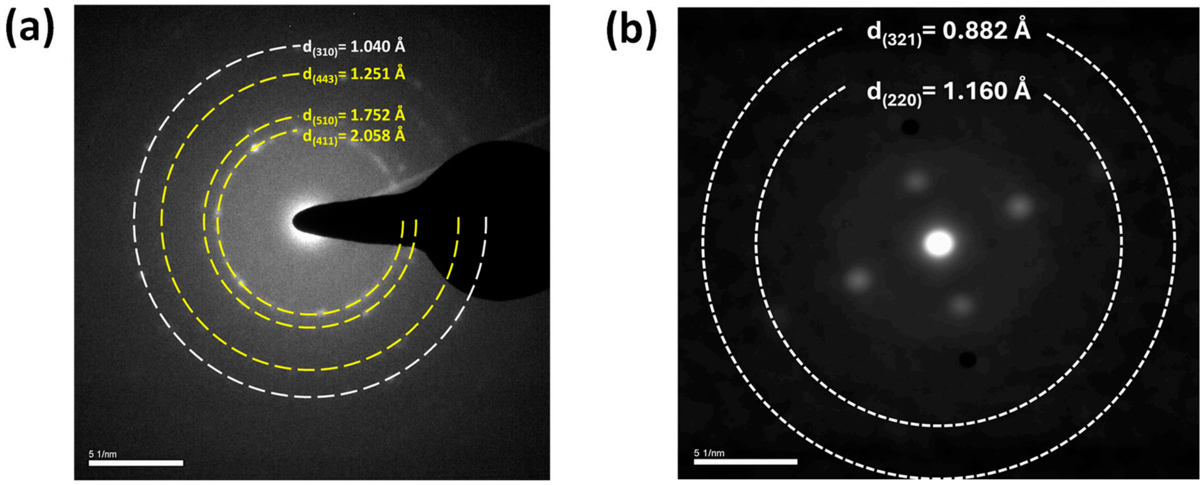

Figure S4. Magnified SAED patterns with indexes for (a) $Cu_{51}Zr_{14}$ (dash yellow curves) and Ta (dash white curve) nanocrystals and (b) Ta nanocrystal for hot pressed BMG composite prepared with an applied pressure of 1.20 GPa.
